# Carboxyalkylated Lignin as a Sustainable Dispersant for Coal Water Slurry

**DOI:** 10.3390/polym16182586

**Published:** 2024-09-13

**Authors:** Hussein Ahmad Qulatein, Weijue Gao, Pedram Fatehi

**Affiliations:** 1 Faculty of Science and Technology, University of Lille, 59000 Lille, France; 2 Green Process Research Centre, Department of Chemical Engineering, Lakehead University, 955 Oliver Road, Thunder Bay, ON P7B 5E1, Canada; wgao@lakeheadu.ca

**Keywords:** coal water slurry, dispersant, lignin, sustainable product, fuel

## Abstract

Coal water slurry (CWS) has been considered a cleaner and sustainable alternative to coal. However, the challenging suspension of coal particles in CWS has created a major obstacle to its use in industry. This study presents a novel approach to enhance the stability and rheological properties of coal water slurry (CWS) through the utilization of carboxyalkylated lignin (CL) as a dispersant. The generated CL samples had high water solubility of around 9 g/L and a charge density of around 2 mmol/g. All CLs were able to stabilize the coal suspension, and their performance decreased due to the increase in the alkyl chain length of carboxyalkylated lignin. Carboxymethylated lignin (CL-1) improved the stability of the coal suspensions with the lowest instability index of less than 0.6. The addition of CLs reduced the contact angle of the coal surface from 45.3° to 34.6°, and the increase in the alkyl chain length hampered its effect on contact angle changes. The zeta potential measurements confirmed that the adsorption of CL enhanced the electrostatic repulsion between coal particles in suspensions, and the zeta potential decreased with the increased alkyl chain length of CLs due to increased steric hindrance. The rheology results indicated that CLs demonstrated shear thinning behavior. This innovative method showcases the affinity of carboxyalkylated lignin to improve the performance of CWS, offering an environmentally friendly alternative for producing a cleaner product, i.e., sustainable coal water slurry, with improved suspension stability.

## 1. Introduction

The increasing global demand for sustainable energy sources has reignited interest in using coal water slurry (CWS) as a greener substitute with reduced environmental emissions and enhanced combustion [1,2]. However, maintaining the stability and homogeneity of CWS poses an ongoing challenge due to the inherent tendency of coal particles to aggregate, impeding the efficient combustion and transportation of CWS. Dispersants, serving as suspension stabilizers that ensure the stability and pumpability of CWS, are pivotal in addressing such a challenge [3]. Many polymers, such as polystyrene sulfonate, humic acids, cellulose derivatives, dextrin, etc., can be used as dispersing agents for coal and other carbon-based materials [4,5]. However, the selection of dispersants is critical, as they must not only effectively disperse coal particles but also meet stringent environmental and economic criteria [6].

In recent decades, considerable attention has been placed on lignin modification and its broadened application usage such as in composite materials, wood industries, polymer composite industries, electrochemical industries, and pharmaceutical and medical industries [7,8]. Lignin, a complex polyphenolic compound found abundantly in plant cell walls, contains functional groups, such as carboxyl and hydroxyl, which enable interactions with coal particles and water molecules. This feature makes lignin a potential candidate for enhancing the stability of CWS. However, the direct use of unmodified lignin as a dispersant is hindered by its polymeric structure and limited water solubility [9]. To overcome these limitations, chemical modification techniques, such as carboxyalkylation, sulfonation, and oxidation, offer a viable strategy to enhance the solubility and efficacy of lignin as a dispersant in CWS [10,11]. For example, carboxyalkylated lignin (CL) is produced by introducing carboxyalkyl groups onto the lignin structure, enhancing its water solubility and amphiphilic properties to improve interactions with coal particles and water. The side chain of CL consists of an anionic head group, serving as a hydrophilic segment, and a hydrocarbon chain, functioning as a lipophilic segment [12]. This modified form of lignin exhibits efficient dispersant properties, mitigating particle aggregation and bolstering the stability of CWS [13]. The use of carboxylic acid groups, instead of sulfonate groups, will also reduce the generation of toxic gases (e.g., SO_2_) when CWS is burned. However, the dispersant efficiency of this new sustainable dispersant for coal water slurry is yet to be studied, which is one objective of this work.

The crucial role of dispersants in dispersing and stabilizing CWS included two aspects. (1) Dispersants could reduce the surface hydrophobicity (i.e., increase the wettability) of coal particles to reduce the interface stress difference between particles and solution so that the particles could be homogeneously dispersed in the slurry, and (2) the adsorption of dispersants could enhance the electronegativity of the coal particles and decrease their aggregation under the effect of electrostatic repulsion and steric hindrance between particles [14,15]. Interestingly, the carboxyalkylation of lignin would yield lignin derivatives with relatively altered sizes [16], which would create dispersants with different sizes. Depending on the size of the dispersant, the dispersant’s adsorbed mass, and thus, the repulsion force generated between particles, may be different. As a new sustainable dispersant, another objective of this work was to understand how lignin-derived dispersants with different alkyl chain lengths, i.e., with relatively different sizes, would impact the dispersion of coal particles in CWS [17].

The overall aim of this study was to systematically examine the influence of carboxyalkylated lignin with different carboxyalkyl side chains as dispersants in CWS. Characterization techniques, including gel permeation chromatography (GPC), various nuclear magnetic resonance (NMR) spectroscopy techniques, and particle charge detector (PCD) analysis, were utilized to compare the molecular weight, stability, surface, and structural alterations in the modified lignin derivatives. This research evaluated the impact of carboxyalkylated lignin on key aspects, such as rheological characteristics, zeta potential, and overall stability of CWS. The results of this work provide insight into the use of a sustainable lignin-derived dispersant to develop a sustainable coal water slurry.

## 2. Materials and Methods

### 2.1. Materials

Acid-washed unmodified softwood kraft lignin (USKL) was obtained from FPInnovations (Pointe-Claire, QC, Canada) [18]. Eisco Anthracite coal specimen was obtained from hBARSCI company (Honeoye Falls, NY, USA). Sodium hydroxide (NaOH, ≥97 wt.%), ethanol (EtOH, 98 wt.%), sodium chloride (NaCl, 99 wt.%), hydrochloric acid (HCl, 38 wt.%), isopropyl alcohol (C_3_H_8_O, 99.8 wt.%), chloroform-D (CDCL_3_), pyridine, 2-chloro-4,4,5,5-tetramethyl-1,3,2-dioxaphospholane, potassium hydroxide (KOH), para-hydroxybenzoic acid, polydiallyldimethylammonium chloride (PDADMAC), sodium chloroacetate (SCA, 98 wt.%), 5-chlorovaleric acid (CVA, ≥98 wt.%), and 11-bromoundecanoic acid (BUA, 99 wt.%) were all obtained from Sigma-Aldrich (Oakville, ON, Canada) and used as received.

### 2.2. Preparation of Coal Water Slurry

Coal samples were obtained in substantial chunks and subsequently subjected to grinding using a high-speed multifunctional grinder as per the ASTM D197 standard test method [19]. The fine ground particles of coal make it easier to blend with other ingredients in CWS preparation and also provide efficiency and environmental performance of coal processing in terms of reactivity, surface area, combustion kinetics, heat quality, emission of pollutants (e.g., SO_2_, NO), and ash content [20,21,22]. This grinding process yielded ground coal with a maximum dimension of 220 μm. To remove particles exceeding 106 μm in size, the sample underwent a sieving process using a standard 140-mesh sieve. The resulting coal sample has a D50 particle size of 90 μm. The analysis of particle size distribution for the coal sample was conducted utilizing the Mastersizer 3000 Malvern Instrument (Worcestershire, UK), which was fitted with a light scattering detector. A 1 wt.% coal sample was meticulously prepared by mixing it with deionized water and subsequently allowing it to stir at a rate of 300 rpm overnight. Following this preparation, the samples were introduced into the Mastersizer for particle size analysis, where three measurements were conducted to obtain the average values.

For the preparation of coal water slurry (CWS) samples, a precise quantity of the segregated coal sample was mixed with deionized water, maintaining a solid concentration of 50 wt.%. The mixing process occurred over 5 min at a rotational speed of 300 rpm, followed by an additional 30 min of stirring at 1000 rpm. Throughout the experiment, the slurry’s pH was consistently upheld at its inherent pH of 8.

### 2.3. Synthesis of CL Samples

**Figure 1:**
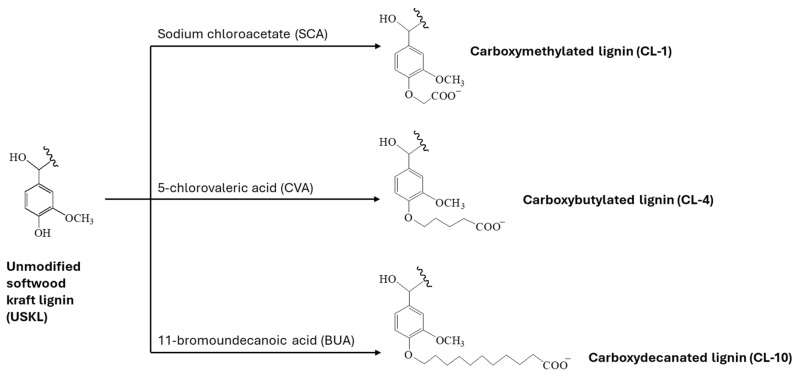
Chemical modifications through carboxyalkylation reactions.

The unmodified softwood kraft lignin (USKL) underwent chemical modifications via carboxyalkylation reactions targeting the phenolic hydroxyl groups present in USKL, as depicted in Figure 1. The process of carboxymethylation of kraft lignin followed previous procedures documented in the literature [13]. Generally, the degree of modification achieved in the carboxyalkylation reaction was adjustable by varying factors, such as the ratio of reactant reagent to kraft lignin.

To synthesize carboxymethylated lignin (CL-1), a solution containing 1 M NaOH was introduced into the lignin solution (at a concentration of 16 g/L) to attain a pH of 12. Subsequently, the SCA reagent underwent a reaction with lignin, maintaining a molar ratio of 4/1 SCA/USKL, and the reaction was conducted at a temperature of 80 °C for 240 min [16,23]. Following the reaction, the mixture was neutralized and then subjected to dialysis for 48 h using cellulose membranes. Similarly, USKL powder was solubilized at pH 12 without the addition of any grafting reagent and was then labeled as the control sample (CS).

Two additional carboxyalkylated lignin variations, specifically carboxybutylated (CL-4) and carboxydecanated (CL-10) lignin, were synthesized following the procedures outlined by previous studies [16,24]. In these experiments, 1.5 g of lignin (USKL) was dispersed in a solution comprising 45 mL of isopropyl alcohol and 12 mL of 30 wt.% NaOH at a temperature of 25 °C, and the mixture was stirred for 30 min. Subsequently, the lignin was subjected to grafting with 5-chlorovaleric acid (CVA/USKL molar ratio of 1/1) and 11-bromoundecanoic acid (BUA/USKL molar ratio of 1/3) at an elevated temperature of 80 °C for 120 min to yield CL-4, and CL-10, respectively. The resulting reaction products were subjected to multiple washes using ethanol/water (at a volume ratio of 40/10) and subsequently recovered through centrifugation. The resultant precipitates were dissolved in 50 mL of deionized water and then purified through a two-day dialysis process. During the dialysis, the dialysate was monitored by UV and conductivity test to verify that the salt and unreacted monomers were completely removed. Subsequent to purification, all products obtained were dried in a vacuum oven set at 60 °C.

### 2.4. Solubility Measurement

For solubility evaluation, 0.2 g of carboxyalkylated lignin (CL) was introduced into 20 mL of deionized water (DW) and subjected to stirring at a speed of 300 rpm for 24 h under ambient conditions. Subsequent to this, each sample underwent centrifugation for 5 min at 1000 rpm, and the resultant supernatant was subjected to overnight drying within a 100 °C oven. The solubility of CL was calculated as described previously [13].

### 2.5. Charge Density and Carboxyl Group Analysis

The Particle Charge Detector manufactured by Mutek, PCD 04, Wessling, Germany, was employed to ascertain the charge density of USKL and CL samples. In this sequence of experiments, a 0.2 g dried sample was introduced in 20 mL of deionized water and allowed to stir for 24 h at room temperature and 300 rpm. Subsequent to this, the samples underwent centrifugation for 5 min at 1000 rpm, and the ensuing supernatants were harvested for the charge density analysis. Then, 1 mL of the supernatant was subjected to titration using PDADMAC (0.0069 M) as a standard cationic solution [16], thereby facilitating the determination of the samples’ charge density.

The quantification of carboxylic acid content in the lignin derivatives was accomplished utilizing an automated potentiometric titrator (Metrohm, 905 Titrado, Switzerland) with a standard solution of 0.1 M HCl employed as the titrant [25]. To initiate this process, 0.06 g of lignin sample was dissolved in a solution containing 100 mL of deionized water, 0.8 M standard KOH, and 0.5 wt.% p-hydroxybenzoic acid. Subsequently, the samples were subjected to titration utilizing a standard 0.1 M HCl solution, thereby enabling the quantification of carboxylic acid content according to the previous study [13].

### 2.6. NMR Analysis

The chemical composition of lignin samples was examined through various NMR techniques, including ^1^H and ^31^P nuclear magnetic resonance (NMR), as well as two-dimensional heteronuclear single-quantum coherence (HSQC) NMR analysis. These analyses were conducted using a Bruker Advance spectrometer (AVANCE Neo NMR-500 MHz instrument, Fällanden, Switzerland) under ambient conditions. To prepare samples for HSQC NMR analysis and ^1^H NMR investigation, approximately 70 mg of lignin samples were dissolved in 1 mL of DMSO-d_6_. The ^31^P NMR study aimed to quantify the aliphatic hydroxyl, phenolic hydroxyl, and carboxyl groups within lignin. This analysis was performed in a CDCl_3_/pyridine mixture using 2-chloro-4,4,5,5-tetramethyl-1,3,2-dioxaphospholane as the phosphorylation reagent and cyclohexanol as the internal standard [16]. To prepare the sample, 70 mg of dried lignin powder was added directly into a clean 20 mL sample vial. Under a fume hood, 1 mL of pyridine/CDCl_3_, 140 µL of relaxing agent, and 70 µL of internal standard were added and set to mix overnight. Prior to running the sample, 200 µL of phosphorylating reagent was added and allowed to mix for a few minutes. The solution was then transferred to an NMR tube using a long-neck Pasteur pipette to load it to the NMR for analysis.

### 2.7. Molecular Weight Analysis

The quantification of the molar mass for the derivatives of lignin was executed using gel permeation chromatography (GPC) employing ultraviolet (UV) and refractive index (RI) detectors. The specific instrument utilized for GPC was the Viscotek GPCmax, manufactured by Malvern (Worcestershire, UK). The GPC analysis employed PolyAnalytic PAA206 and PAA203 columns with a 0.1 mol/L NaNO_3_ solution as the eluent, flowing at a rate of 0.7 mL/min. The analysis transpired at a temperature of 35 ℃. For calibration purposes, a reference standard of polyethylene oxide was employed [16]. Roughly 50 mg of dried CL samples were solubilized in a solution encompassing 10 mL of eluent solution a day prior to the test. The resulting solutions were then filtered using a 0.2 µm pore size nylon filter with a 13 mm diameter. Following that, the filtered solutions were used to calculate molecular weight.

### 2.8. Zeta Potential Analysis

The zeta potential of coal slurries was determined in the presence and absence of CL utilizing a NanoBrook Zeta PALS instrument (manufactured by Brookhaven Instruments Corp, Nashua, NH, USA). In this experiment, 15 g of coal powder was introduced into equal amounts of either deionized water or a lignin solution at a specified concentration. This mixture was then allowed to stir at room temperature for 30 min before being transferred to an electrophoretic cell for zeta potential measurement. Each experiment was replicated five times, and the resultant mean values are presented in this study.

### 2.9. Contact Angle Analysis

An optical tensiometer, Theta lite from Biolin Scientific, Espoo, Finland, which was equipped with a camera, was employed to assess the wettability of coal samples having varying particle sizes. In this analysis, coal samples were prepared at 50 wt.% concentration. Subsequently, several microscopic glass slides were dipped in coal sample mixture for 2 s to coat the surface and then subjected to oven drying overnight. This drying process occurred at 60 °C. To determine the contact angle between deionized water and the coal samples coated on the slides, the sessile drop method was utilized, with approximately 7 microliters of water droplets placed on the coated slides. This contact angle measurement was executed using the optical tensiometer [26]. In a separate set of experiments, a droplet (7 µL) of 0.1 wt.% CL solutions was deposited onto the coal-coated slides, and the contact angle between the prepared droplets and the coal-coated slides was measured using the same procedure as described earlier. Each experiment was repeated three times, and the average results were determined.

### 2.10. Viscosity Analysis

The determination of the apparent viscosity of the slurry was executed utilizing the American-made Brookfield DV-II + Pro rotary viscometer (Middleborough, MA, USA). The operational principle of this rotary viscometer involved propelling a spindle submerged in the test fluid through a calibrated spring. The spring’s deflection was utilized to gauge the viscous resistance of the fluid against the spindle, and the deflection was measured through a rotary transducer. The measurements were conducted at a consistent temperature of 25 °C employing the LV-4 spindle. To elucidate the shear-dependent rheological characteristics of the samples, the shear rate was varied across a range from 0 s^−1^ to 190 s^−1^, and the subsequent viscosity was recorded as a function of the shear rate. The apparent viscosity values of CWS were recorded by the viscometer at a constant shear rate of 100 s^−1^ and room temperature.

### 2.11. Stability Analysis

The stability evaluation of the CWS samples was conducted utilizing a vertical scan analyzer called Turbiscan Lab Expert developed by Formulaction, Toulouse, France. The Turbiscan Lab Expert employed laser light with an 880 nm wavelength to probe the transmittance and backscattering characteristics of suspensions. The total stability index (TSI) represented the cumulative sum of changes in backscattering or transmission across the entire sample caused by destabilization. Thus, a higher TSI indicated greater instability within the sample. These measurements serve to assess the stability of the investigated suspensions. The data acquired from the instrument, encompassing the measurements of both transmission and backscattering, were gathered at intervals of 25 s over one hour, followed by hour-long intervals for the subsequent 24 h. The gathered data were subsequently subjected to analysis using Turbisoft software (version 2.1, Formulaction, Toulouse, France).

Within one series of experiments, 30 mL of the CWS at a 50 wt.% concentration was introduced into cylindrical tubes, and the instrument subsequently scrutinized the stability of the CWS. In another series of experiments, a CWS possessing a concentration of 50 wt.% was prepared in the presence of 0–2 wt.% CL. Changes in the suspension stability were then meticulously tracked by the instrument at 25 °C.

## 3. Results and Discussion

### 3.1. Properties of Coal and Lignin Samples

Table 1 tabulates an overview of the charge density, solubility, molecular weight (MW), polydispersity index (PDI), and carboxylic acid content of lignin derivatives. As previously mentioned, CL was synthesized after attaching a carboxylate group to the phenolic hydroxyl group of USKL (Figure 1). USKL exhibited a lower anionic charge density of 0.35 mmol/g and a carboxylate group content of 0.63 mmol/g. In contrast, the CL samples displayed a high anionic charge density of around 2 mmol/g with a higher carboxylate group content of around 1.2 mmol/g, confirming the success of the carboxyalkylation reaction. The results also indicate that the aliphatic hydroxy groups of USKL can be substituted with carboxylate groups in the carboxyalkylation reactions [24,27]. Additionally, the control lignin sample (CS) had a carboxylate group content of 0.9 mmol/g and anionic charge density of 1.3 mmol/g, i.e., higher than those of USKL. This increased value could be attributed to the partial oxidation of the OH groups of USKL when undergoing all the process conditions described in Section 2.3 [28]. The molecular weights (MWs) of the prepared samples reported in Table 1 show an inclining sequence, with CL-1 having the lowest molecular weight of other samples. This could be due to the additional weight contribution from the grafted chains after the carboxyalkylation reactions to produce the lignin derivatives [23,29]. The variation in the polydispersity index of lignin derivatives might stem from the incorporation of carboxyalkylate groups into the lignin structure [30].

In contrast to USKL, all lignin samples were found to have higher solubility values of around 9 g/L. The enhanced solubility can be attributed to the heightened favorability of nucleophilic substitution, where the OH group is replaced by a carboxylate group in the para position on the lignin’s phenolic ring [31]. Additionally, the increased solubility of the CS is due to the enhanced hydrophilicity from the deprotonation of the phenolic group, which causes stronger interactions with water molecules [32].

### 3.2. NMR Spectroscopy

**Figure 2:**
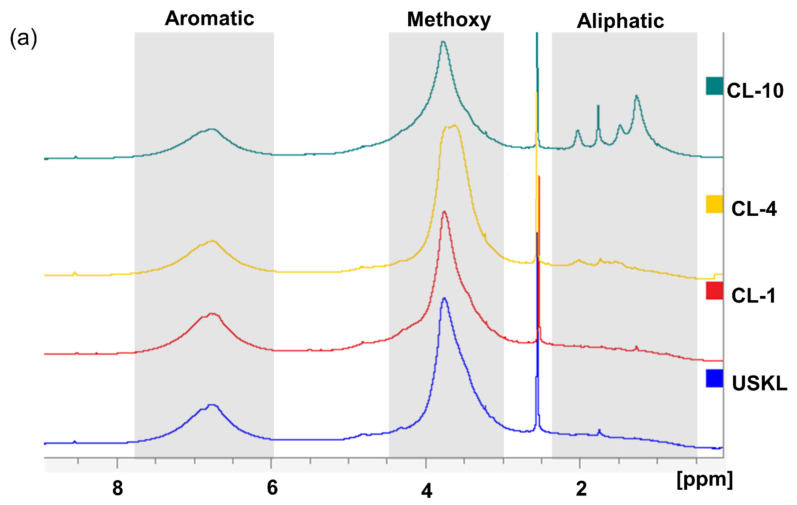

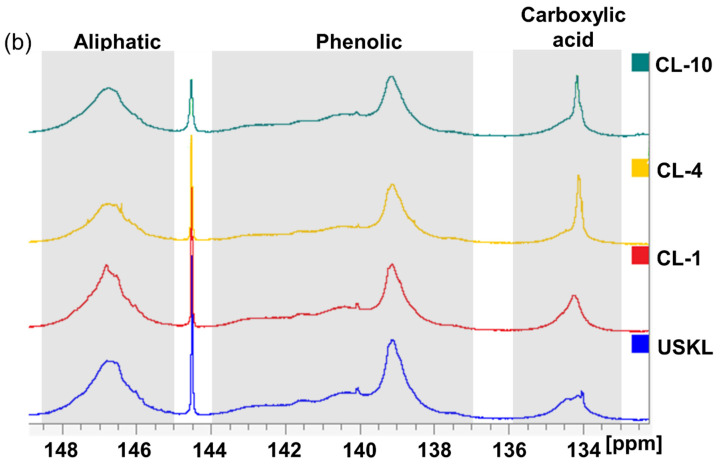
^1^H NMR spectra (**a**) and ^31^P NMR spectra (**b**) of the lignin derivatives.

Figure 2a shows the ^1^H NMR spectra of the lignin derivatives. The methoxy (-OCH_3_) and aromatic groups are represented by wide signal peaks in the ranges of 3.0 to 4.5 ppm and 6.0 to 7.8 ppm, respectively [13,33]. Notably, the presence of the DMSO-d_6_ solvent generated a peak at 2.50 ppm [34]. Following the carboxyalkylation procedure, distinct new peaks in the aliphatic region in the range of 0.5–2.4 ppm became noticeable, indicating the presence of protons from the grafted alkyl chains. This demonstrates the incorporation of alkyl side chains with carbon numbers of 1, 4, and 10 into the lignin framework for CL-1, CL-4, and CL-10, respectively. Based on the overall results obtained from the ^1^H NMR analysis, it can be concluded that the grafting reactions involving all three types of reagents were successfully executed to enhance both their water solubility and amphiphilic properties [16].

Figure 2b shows the ^31^P NMR spectra of lignin derivatives, while Table 2 provides their quantitate hydroxyl group contents. A noticeable reduction in the signal strength linked to phenolic hydroxyl groups in the spectra of the carboxyalkylated samples can be seen after the lignin underwent carboxyalkylation, particularly affecting signals linked to H-, G-, and C5-substituted OH. As a result, the samples’ phenolic hydroxyl group concentration dropped from 4.48 mmol/g for USKL to 2.90 mmol/g, 2.42 mmol/g, and 2.89 mmol/g for CL-1, CL-4, and CL-10, respectively. However, only little changes were seen in the aliphatic hydroxyl groups, indicating that the phenolic hydroxyl groups were the primary target of the S_N_2 reaction, which mostly affected the lignin structure [35]. In line with expectations, the addition of alkyl chains significantly increased the peak intensity of carboxyl groups, causing the amount of carboxylic OH groups to rise from 0.76 mmol/g in USKL to 1.06 mmol/g for CL-1, confirming the success modification reaction. It is noteworthy that the prominent peak at 144.50 ppm corresponds to the presence of the internal standard [36].

**Figure 3:**
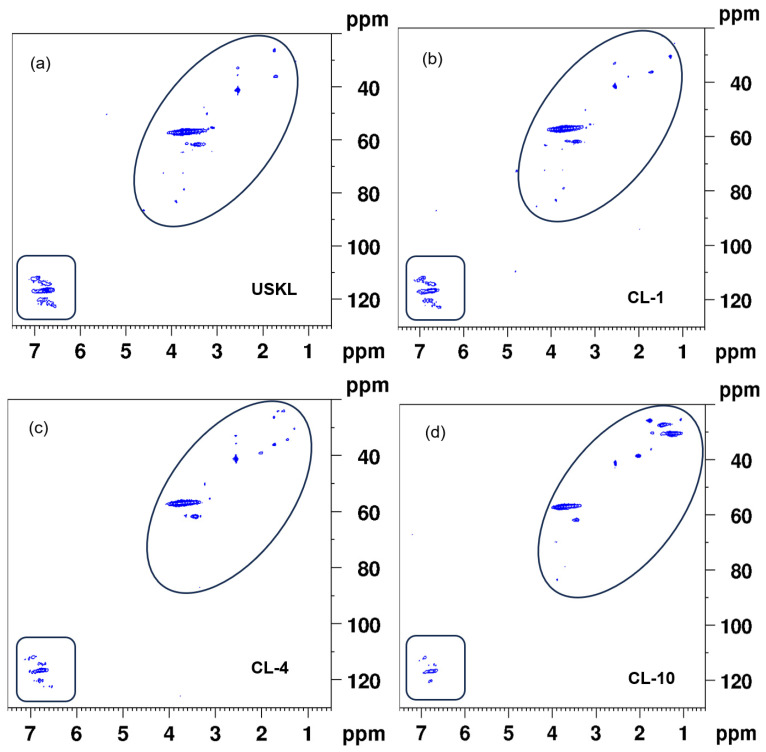
HSQC NMR spectra of lignin samples: (**a**) USKL; (**b**) CL-1; (**c**) CL-4; (**d**) CL-10.

HSQC NMR analysis was performed to elucidate the structural properties of lignin derivatives (Figure 3), which provided insights into the connections and constituent components of lignin. These spectra can be classified into two major regions: the aromatic area (with C/H values ranging from 90 to 130/6 to 7.5 ppm) and the C-C aliphatic region (with C/H values ranging from 20 to 90/0.5 to 5 ppm). The C-C aliphatic region also includes the C-O aliphatic region (with C/H values ranging from 50 to 90/2.75 to 5 ppm). It is worth mentioning that the intensities within the C-C aliphatic region (marked with an elliptical shape) showed an increase, which aligns with the findings from quantitative ^31^P-NMR and ^1^H-NMR analyses.

Within the aromatic region, noticeable correlations linked to guaiacyl (G) units became apparent. In particular, strong correlations were observed for C2/H2, C5/H5, and C6/H6, with corresponding chemical shift values of 112.8/6.8, 116/6.7, and 120/6.7 ppm, respectively (Figure 3) [37]. Additionally, it is worth mentioning that the intensities within the aromatic region (marked with a rectangular shape) showed a decline, which aligns with the findings from quantitative P-NMR and H-NMR analyses.

### 3.3. Stability Studies

**Figure 4:**
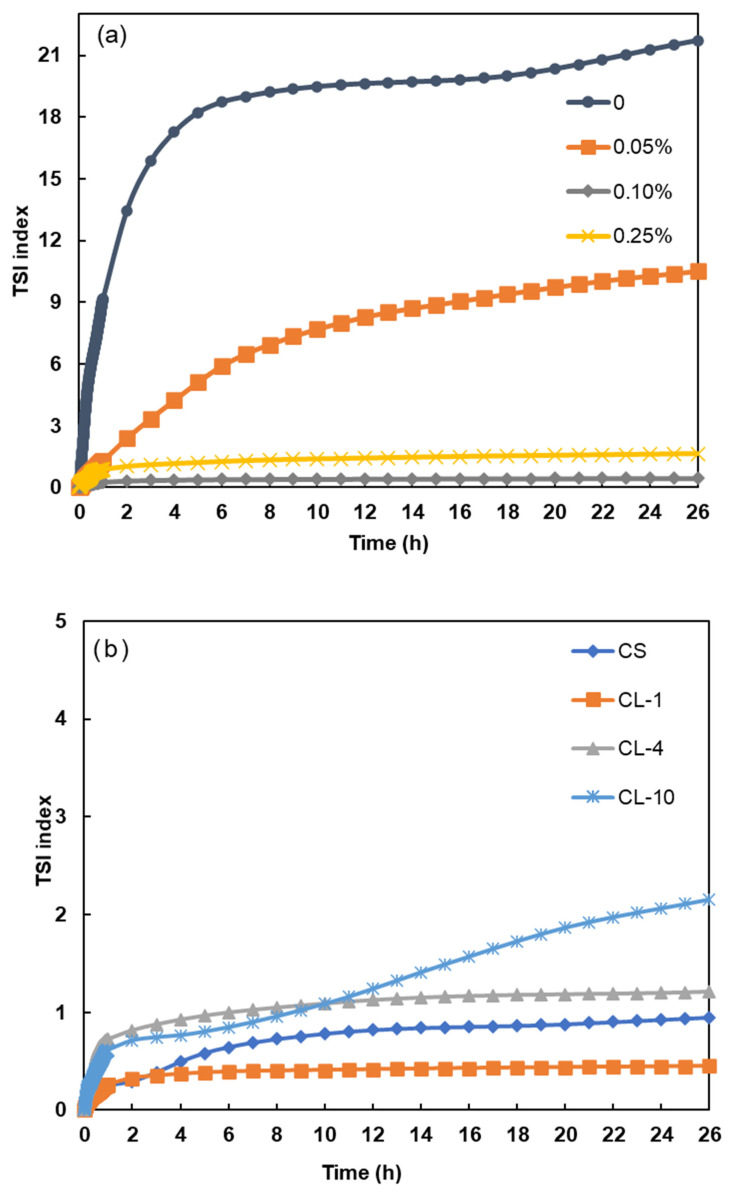
TSI index of suspensions (50 wt.%) (**a**) at different CL-1 dosages and (**b**) in the presence of different CLs (at 0.1 wt.% dosage).

Figure 4a represents the effect of lignin derivatives and dosage on the stability of coal suspension. To test the stability of the samples, CL-1 was chosen to be tested first due to its simpler preparation procedure and higher accessibility to coal particles because of its shorter alkyl chain length. CL-1 was able to stabilize the coal suspension at all dosages (Figure 4a and Figure S1). Based on the transmission (ΔT%) and backscattering (ΔBS) profiles data (Figure S1), the clarification and sedimentation kinetics for the dosages of 0.05% were calculated to be 2.77 %T/h and 0.242 mm/h, respectively, which were lower than those (clarification kinetics of 3.385 %T/h and sedimentation kinetics of 0.308 mm/h) in the absence of the dispersant [38]. No clarification and sedimentation kinetics can be calculated for the higher dosages of 0.1% and 0.25% because no sedimentation was observed, indicating satisfactory dispersion. It can be seen in Figure 4a that the 0.1 wt.% CL-1 provided the lowest instability index. Thus, this dosage was selected to test the performance of the other two CL samples as dispersants for coal suspension. This could be attributed to charge interactions between the prepared dispersant’s surface and that of the coal particles. The higher instability index at 0.05 wt.% CL-1 dosage may be attributed to an insufficient quantity of CL-1 adsorbed to the coal particles to cover their surface [39]. As the dosage increased to 0.1 wt.%, the dispersion effect of CL-1 became more significant by surrounding the coal particles, keeping the coal particles from coming close to each other via electrostatic repulsion, which was confirmed by enhanced zeta potential in Figure 4a. The slightly higher instability index at a higher dosage of 0.25 wt % than that at 0.1 wt.% may be due to excess CL-1, which can get entangled with the CL-1 chains/branches not entirely adsorbed to coal particles, causing coal particles to agglomerate/flocculate. Previous reports have suggested that excessive amounts of dispersant increase the ion concentration, causing compression of both the adsorbed dispersant layer and the electric double layer, leading to particle agglomeration [40].

Figure 4b depicts the effects of lignin derivatives as dispersants (0.1 wt.%) on the stability of the coal suspension. It can be seen that the addition of all lignin samples improved the stability of the coal suspension as the instability index decreased to less than 2. Among all the CLs, CL-1, with the shortest alkyl chains, achieved the lowest instability index of less than 0.6. No clarification and sedimentation kinetics can be calculated for CL-1 and CL-4 at dosages of 0.1%, as no sedimentation was observed. CL-10 had the highest clarification (0.731 %T/h) and sedimentation (0.207 mm/h) kinetics among all the lignin derivatives, demonstrating that CL-10 has physicochemical properties that reduced its performance for an effective dispersion of coal slurry (Figure S1). Considering the similar solubility and charge density of the lignin derivatives, the main difference in their performance is their grafted alkyl chain length and, therefore, molecular weight. Compared to CL-1 and CL-4, the longest alkyl chain length of CL-10 could lead to lower interactions for coal particles due to steric hindrance and thus reduced dispersion performance [3]. It was also observed that the instability indices of CL-10 and CL-4 were comparable up to 4 h, while a gradual increase for CL-10 was observed after 4 h. The phenomenon could be attributed to the relatively higher hydrophobic association tendency of CL-10 induced by its long alkyl chain, leading to the slow agglomeration of the coal particle over time [41].

### 3.4. Zeta Potential of Coal Particles

**Figure 5:**
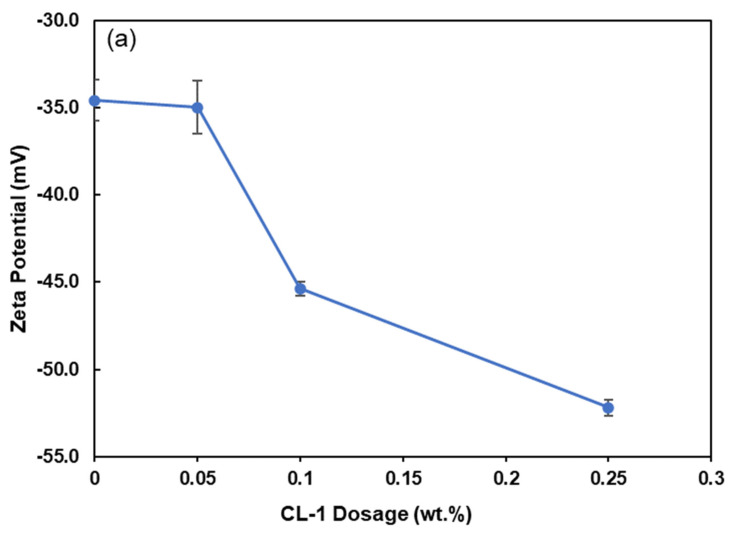

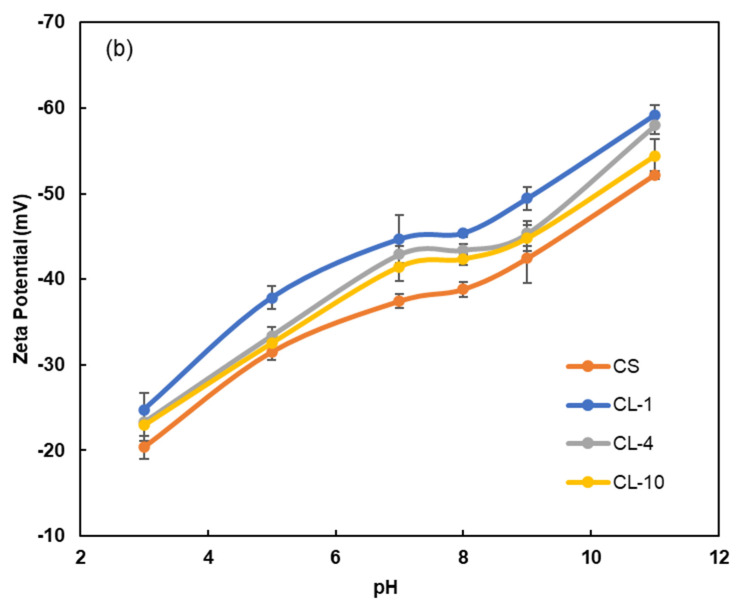
Zeta potential of coal suspensions (50 wt.%) as a function of (**a**) CL-1 dosage and (**b**) pH (at 0.1 wt.% dosage).

Understanding the zeta potential of coal particles in coal water suspensions is crucial for revealing the interaction mechanism between the dispersant and coal particles. Factors such as coal properties, dispersant type, and anionic ions in the solution influence the zeta potential of suspensions [42]. Figure 5 depicts the zeta potential of the coal suspension in the presence of CL-1, while coal loading was maintained at 50%. In the absence of the dispersant, the zeta potential of coal particles was measured at −34.6 mV, implying that the original coal particles carried a negative charge [43]. In the presence of CL-1, the zeta potential dropped to −52 mV, which is attributed to the attachment of CL-1 and lignin’s carboxylic acid group developing more anionic charge groups on the coal surface [39]. Therefore, the improved stability of coal particles can be attributed to electrostatic repulsion mechanisms that were raised as a result of lignin adsorption onto the particle surfaces [10].

Figure 5b shows that the zeta potential of all lignin derivatives increased as pH increased from 3 to 11 because of the deprotonation of carboxyl and phenolic hydroxyl groups [44]. The pKa of grafted -CH_2_COOH (4.75) for CL-1, -(CH_2_)_4_COOH (4.84) CL-4, and -(CH_2_)_10_COOH (4.89) for CL-10 slightly increased with the alkyl chain length [45,46,47], leading to a slightly faster increase in the zeta potential of CL-1 at pH 5. CS had the lowest effect on zeta potential due to its lowest charge density (Table 1). In practice, the pH of the CWS is usually between 6 and 8 [48,49]. Comparing all the CL samples at this pH range, CL-1 exhibited superior performance in enhancing the zeta potential of coal particles in the range of pH 7 and 8, where the zeta potential charge reached approximately −45.4 mV, causing more repulsion between the coal surfaces and better dispersion performance (Figure 5b). The decreased zeta potential from CL-1 to CL-10 is possibly due to the enhanced steric hindrance of CLs with the increased alkyl chain length, resulting in the reduced adsorption of CLs on the coal surface [3].

### 3.5. Contact Angle Studies

**Figure 6:**
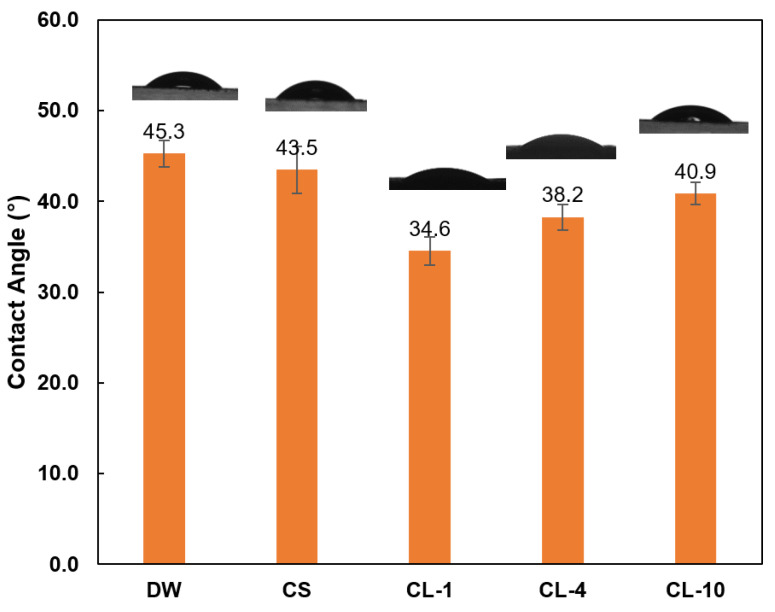
Contact angle of water, CS (control sample), and CL (0.1% solution) on the surface of coal particles.

The contact angle of water droplets containing 0.1 wt.% lignin derivatives on a surface covered by the coal sample is presented in Figure 6. The results confirmed that the addition of lignin derivatives reduced the contact angle from 45.3° to 34.6° for CL-1. The major region of the coal particle surface is hydrophobic [49]. The adsorption of CL polymer on the surface of coal particles could mask the hydrophobic sites and render the surface hydrophilicity, which improves the coal–water interaction and weakens the hydrophobic association among coal particles. Also, the increase in the alkyl chain length gradually increased the contact angle, which is attributed to increased carboxylation chain length with higher hydrophobicity.

According to the extended Derjaguin–Landau–Verwey–Overbeek (DLVO) theory of colloid stability, the degree of particle dispersion/aggregation is governed by a balance between repulsive (electrostatic, hydration, steric) and attractive (van der Waals, hydrophobic) interparticle forces [4]. Therefore, the longer alkyl chain with higher hydrophobicity would increase the attractive interparticle forces, resulting in more particle aggregation and less suspension stability. This is consistent with the stability studies for CL-1, CL-4, and CL-10 in Figure 4b.

### 3.6. Viscosity Studies

Generally, coal particles disperse effectively within the water medium if they do not adhere to themselves. The presence of water molecules between the coal particles acts as a lubricant, facilitating the movement of particles when particles attempt to collide [50]. However, with the introduction of a dispersant, a subtle alteration in the behavior of the CWS can be observed. At this stage, the slurry demonstrates characteristics akin to a shear-thinning fluid [51]. The surfactant addition alters the rheological characters of these slurries from non-Newtonian towards Newtonian fluids [52], which typically occurs at a solid concentration of around 50%, marking the onset of particle overcrowding.

**Figure 7:**
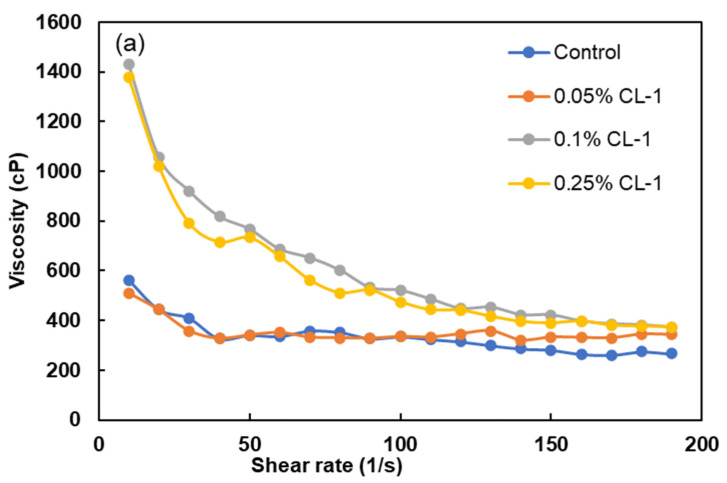

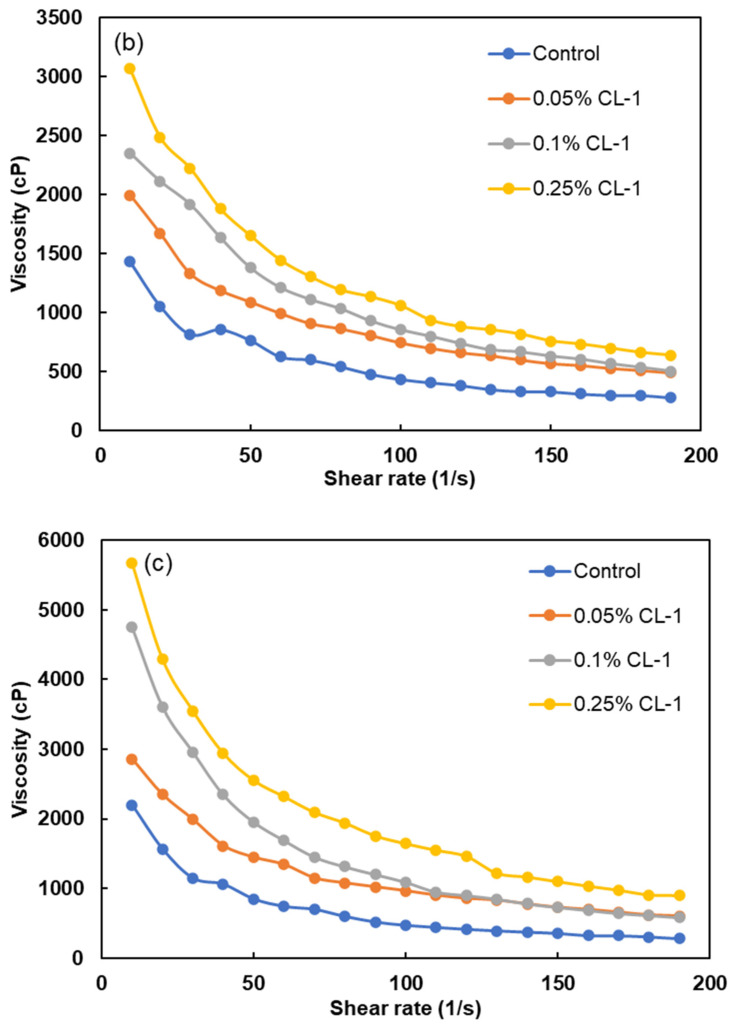

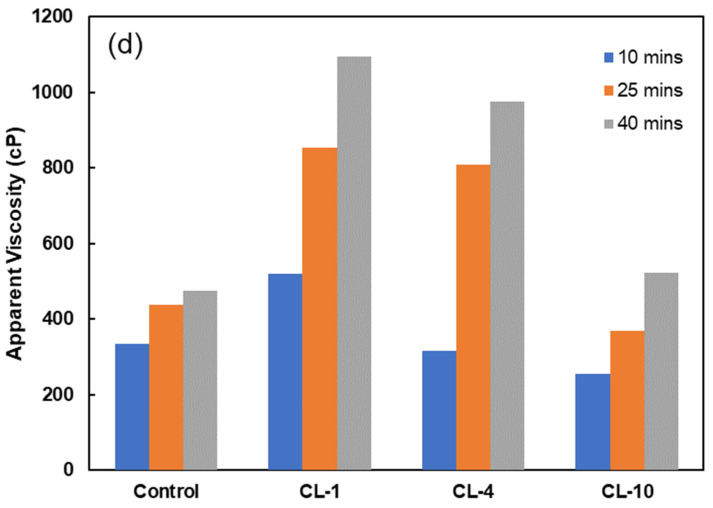
Viscosity vs. CL-1 dosage (**a**) after 10 min, (**b**) after 25, (**c**) after 40 min, and (**d**) apparent viscosity of CWS as a function of dispersant types (CL-1 dosage: at 0.1 wt.%; solid concentration of coal suspension: 50 wt.%.

Consequently, the coal particles begin to directly interact with each other in the absence of sufficient water molecules due to reduced water availability. This shift results in coal–coal shearing taking precedence over coal–water shearing, leading to the manifestation of apparent viscosity [53]. The overcrowding of particles prompts the formation of a compact and robust structure within the slurry, making it resistant to disruption. Furthermore, at higher shear rates, the CWS continues to display shear-thinning behavior [54]. The apparent viscosity of CWS was determined by varying the shear rate of CWS in the presence of 0–0.25 wt.% CL-1. Figure 7a–c represent these effects on the apparent viscosities after 10 min, 25 min, and 40 min, respectively. It can be observed that by increasing the shear rate at each time point, the apparent viscosity of CWS rapidly dropped, demonstrating shear thinning behavior (Figure 7a–c). The figures also show that the apparent viscosity increased as the storage time extended. The changes in viscosities over time can be attributed to the interaction between water and coal, resulting in the filling of its pores and reducing the amount of available water between particles [50]. Consequently, this impedes the movement of particles and leads to an elevation in viscosity [55]. It can also be observed that apparent viscosity exhibits an increasing trend with more addition of dispersants after 10 min, 25 min, and 40 min, consecutively (Figure 7a–c). This can be explained by dispersant–dispersant and/or dispersant–water interactions due to the free-floating dispersant molecules [56].

Furthermore, the viscosity analysis at a low shear rate is commonly used to assess dispersion stability. Based on the data presented in Figure 7, more variations in viscosity were observed at a lower shear rate than at a higher shear rate over different time points. This phenomenon could be due to the extended time required for the dispersion to stabilize, which can take up to 24 h for some dispersants. The findings indicated that among all of the prepared samples, CL-10 had the best effect on lowering the viscosity of the CWS prepared in coal (Figure 7d). These results complement the results presented in the contact angle and zeta potential sections. The reason behind this phenomenon lies in the higher hydrophobicity of the surface of coal in the case of higher rank coals used in this study. This, in turn, leads to an increased level of hydrophobic–hydrophobic interactions that occur between the coal surface and the hydrocarbon chain of the dispersant [55].

For CWS to be an effective fuel, it should have a high coal content for addressing the energy demand and high stability and long shelf life for its long-term storage, long-distance transportation, and the processing of the slurry (e.g., mixing, pouring, and general handling) [57,58]. CWS with good stability often has a ‘shear thinning’ behavior, which keeps a high viscosity in a static state and a low viscosity during transportation [57]. Compared to the control sample, the addition of CL dispersant enhanced the shear thinning behavior of CWS by increasing viscosity in a static state (at a shear rate of 0) and reducing viscosity at higher shear rates (Figure 7), indicating the desirable rheological properties for serving as a good fuel source with appropriate storage, transportation, and processing properties. More in-depth rheological analysis (e.g., oscillation) will be continued in our future work to discuss how the addition of a dispersant would modify the slurry.

### 3.7. Mechanism of Dispersion

**Figure 8:**
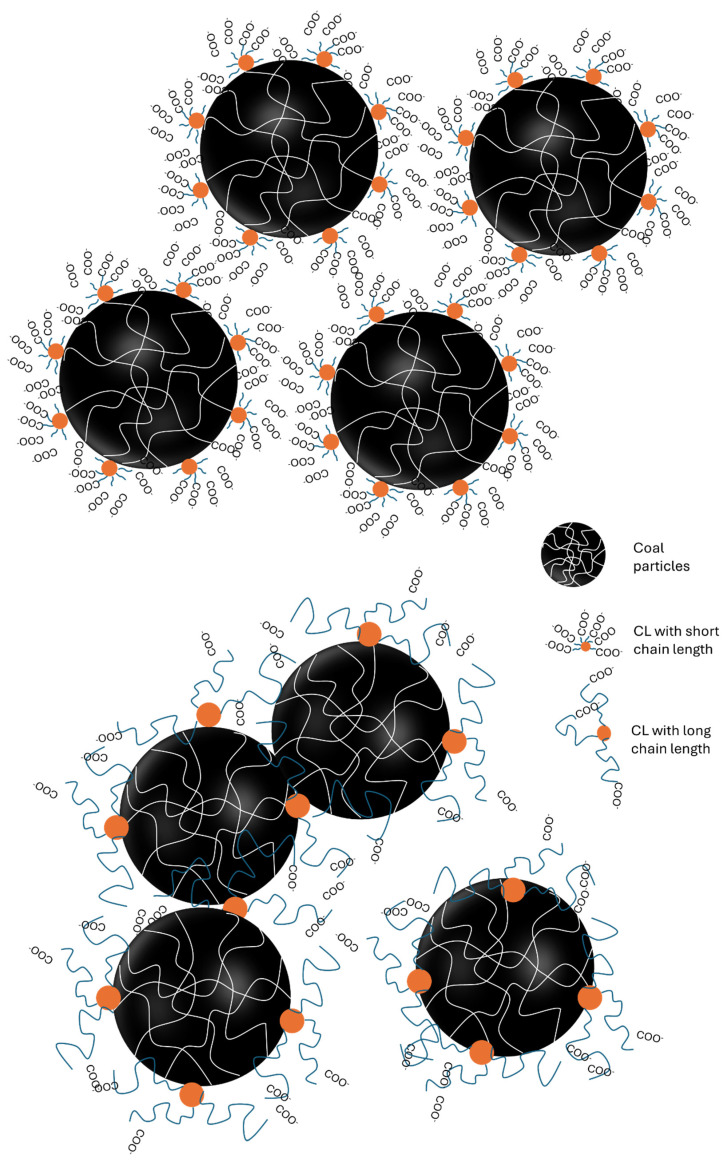
Schematic representation of coal particles dispersed by carboxyalkylated lignin with different alkyl chain lengths.

Figure 8 presents a schematic representation of stabilizing coal particles by carboxyalkylated lignin-based dispersants with different alkyl chain lengths in an aqueous medium. Based on the zeta potential (Figure 5) and contact angle (Figure 6) results, it is suggested that the CL dispersant with a short alkyl chain (e.g., CL-1) tends to adsorb on the hydrophobic coal surface via its hydrophobic head, orienting the hydrophilic carboxyl tail diffusing into water and providing more negative surface charges. Therefore, the particles could be homogeneously dispersed in the slurry mainly through two mechanisms. (1) Enhanced surface negative charges provided electrostatic repulsion between adjacent coal particles, and (2) the surface hydrophilicity of coal particles was enhanced for lubricating and reducing the interface stress between particles and water (Figure 8). However, in the case of CL dispersant with a long alkyl chain (e.g., CL-10), the steric hindrance of the long chain reduced its adsorption on the coal surface and resulted in less negative surface charge for stabilizing the particles (Figure 5). Furthermore, the long alkyl chain had a higher hydrophobic association tendency, leading to particle collision aggregation through hydrophobic–hydrophobic interparticle attractions.

### 3.8. Comparison

The carboxyalkylated lignin for the CWS application generated in this study is compared with other lignin-based dispersants reported in the past in Table 3. As lignin cannot dissolve in neutral water, most of these studies were focused on lignosulfonate or sulfonated alkali lignin to increase the water-soluble property and dispersing ability in CWS. Interestingly, the carboxyalkylated lignin generated as a sustainable dispersant does not have a sulfonate group; thus, its contribution to SO_2_ emissions will be very limited when it is burned along with CWS, which is very advantageous. It can be seen that CL polymer achieved relatively lower CWS viscosity at the lowest dosage compared to other lignin-based dispersants. This behavior could be attributed to its high solubility and charge density, which effectively increased the hydrophilicity on the surface of the coal particles. The molecular weight of CL was also much lower, which facilitated its accessibility to the coal surface with small-sized molecules. At an industry-scale production, the concentration of CWS with an apparent viscosity of 1200 cP is defined as the maximum slurry concentration [59]. Although the CWS solid concentration used in this study was relatively lower than the others, considering its low dosage and viscosity, a higher concentration of CWS can be potentially used to satisfy its combustion/gasification application further.

## 4. Conclusions

The carboxyalkylation process was proven to successfully incorporate carboxyalkyl groups with various chain lengths into lignin structure, which effectively enhanced the water solubility, charge density, and dispersion capabilities of lignin. Carboxymethylated (CL-1) lignin achieved better stability of the coal suspensions than carboxybutylated (CL-4) and carboxydecanated (CL-10). All the CL samples improved the stability of coal particles via electrostatic repulsion mechanisms due to their adsorption onto the coal particle surfaces and enhancement in zeta potential. The stability of the coal suspension decreased with the decrease in the alkyl chain length of CLs. The contact angle results confirmed that the addition of CLs slightly improved the water interaction of coal particles. Still, the increase in the alkyl chain length increased the contact angle gradually. The zeta potential decreased with the increased alkyl chain length, implying the reduced adsorption of CLs on the coal surface due to steric hindrance of CLs. The resulting carboxyalkylated lignin (CL) exhibited improved rheological properties, particularly in terms of apparent viscosity, which greatly contributes to the overall stability and pumpability of CWS. Overall, carboxyalkylated lignin offers a sustainable solution as a dispersant for CWS, utilizing a sustainable dispersant to improve the dispersion of coal water slurry.

## Figures and Tables

**Table 1:** Physiochemical characteristics of lignin derivatives.

Sample ID	Charge Density (mmol/g)	Solubility (g/L)	M_W_ (kg/mol)		PDI		Carboxylic Acid Content (mmol/g)
			RI	UV	RI	UV	
USKL	−0.35 ± 0.02	2.7	-	-	-	-	0.63
CS	−1.34 ± 0.02	9.9	1.50	1.79	1.95	2.14	0.93
CL-1	−1.95 ± 0.03	9.9	1.61	2.12	2.15	2.21	1.17
CL-4	−2.04 ± 0.01	9.6	2.09	2.20	2.04	2.22	1.22
CL-10	−1.98 ± 0.02	9.9	2.31	2.58	1.69	1.87	1.20

Data are not available due to limited solubility.

**Table 2:** Hydroxyl group contents of prepared samples from ^31^P NMR spectrum (mmol/g).

Sample	C5 Substituted	Guaiacyl	p-Hydroxyphenyl	Total Phenolic OH	Carboxylic Acid OH	Aliphatic OH
USKL	2.27	1.95	0.26	4.48	0.76	1.90
CL-1	1.42	1.33	0.15	2.90	1.06	2.04
CL-4	1.08	1.20	0.15	2.42	1.01	1.42
CL-10	1.44	1.29	0.16	2.89	1.15	1.88

**Table 3:** Use of lignin-based dispersants for CWS.

	Mw, kg/mol	Charge Density, mmol/g	Dosage, %	CWS Concentration, %	Contact Angle, °	Zeta Potential, mV	Viscosity at 100 s^−1^, cP	Reference
CL-1	1.6	−1.95	0.1	50	34.6	−45.4	520	This study
Sodium lignosulfonate	10	2.34 *	1	65	74.4	-	632	[59]
Sodium lignosulfonate	9	1.12 *	0.4	67	-	−52	600	[43]
Crosslinked lignosulfonate	42.8–115	1.16–1.31 *	0.6	60	-	-	536–639	[60]
Lignosulfonate	9	-	1	60	-	−28	624–857	[61]
Grafted sulfonated alkali lignin	31.5	2.52 *	1	61.5	72	−65	650	[11]
β-Cyclodextrin grafted alkali lignin	-	-	0.5	55–59	-	−50~−62	1000	[62]

* Sulfonate group content (mmol/g) for lignosulfonate dispersant.

## Data Availability

Data are contained within the article or Supplementary Material.

## Supplementary Materials

The following supporting information can be downloaded at: https://www.mdpi.com/article/10.3390/polym16182586/s1, Figure S1: Transmission (ΔT%) and backscattering (ΔBS) profiles at CL-1 dosages of (a) 0, (b) 0.05 wt. %, (c) 0.10 wt.%, (d) 0.25 wt.%. And transmission (ΔT%) and backscattering (ΔBS) profiles in the presence of (e) CL-4 and (f) CL-10 (at 0.1 wt.% dosage).
