# Supplementary material for: Carboxyalkylated Lignin as a Sustainable Dispersant for Coal Water Slurry
Source: Polymers (Basel). 2024 Sep 13;16(18):2586. doi: 10.3390/polym16182586 (PMC11435015; doi:10.3390/polym16182586)
Supplement: Supplementary file 1 [file polymers-16-02586-s001.zip › polymers-3163879-supplementary.pdf]

Supplementary Materials

Carboxyalkylated lignin as a sustainable dispersant for coal water slurry

Hussein Ahmad Qulatein [a,b], Weijue Gao [b], Pedram Fatehi [b, *]

[a]BioRef European Master Program, University of Lille, 59000 Lille, France

[b]Green Process Research Centre, Lakehead University, 955 Oliver Road, Thunder Bay, Ontario, P7B5E1, Canada

*Corresponding author: pfatehi@lakeheadu.ca

(a)

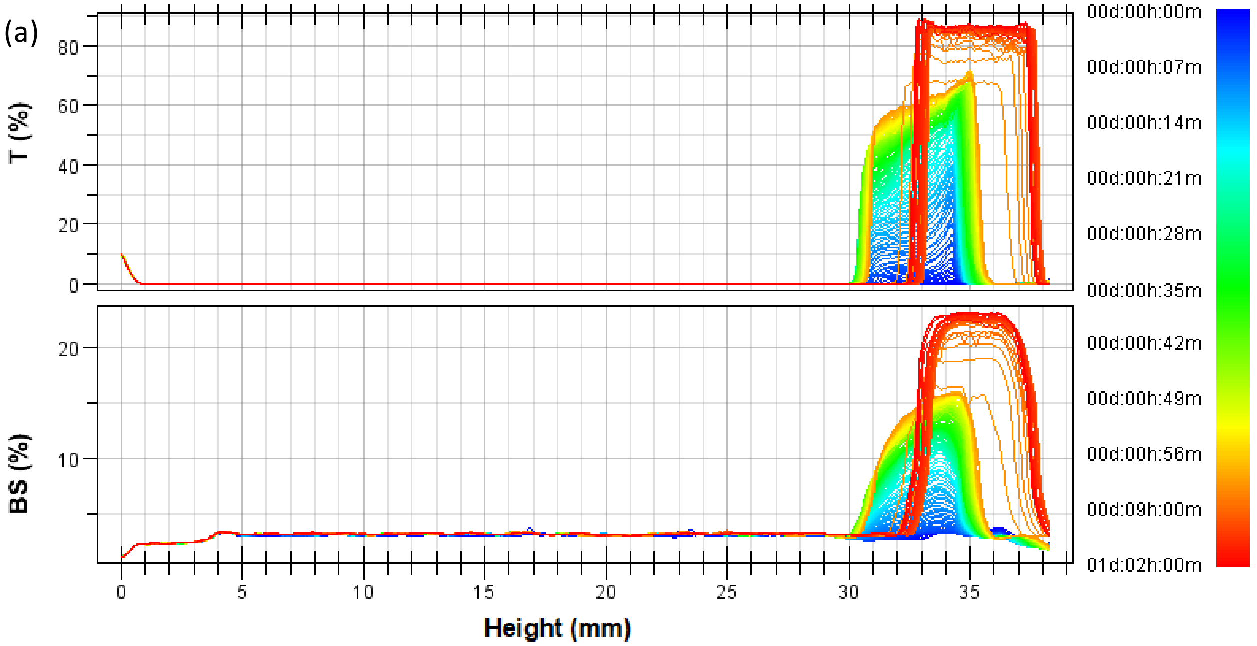

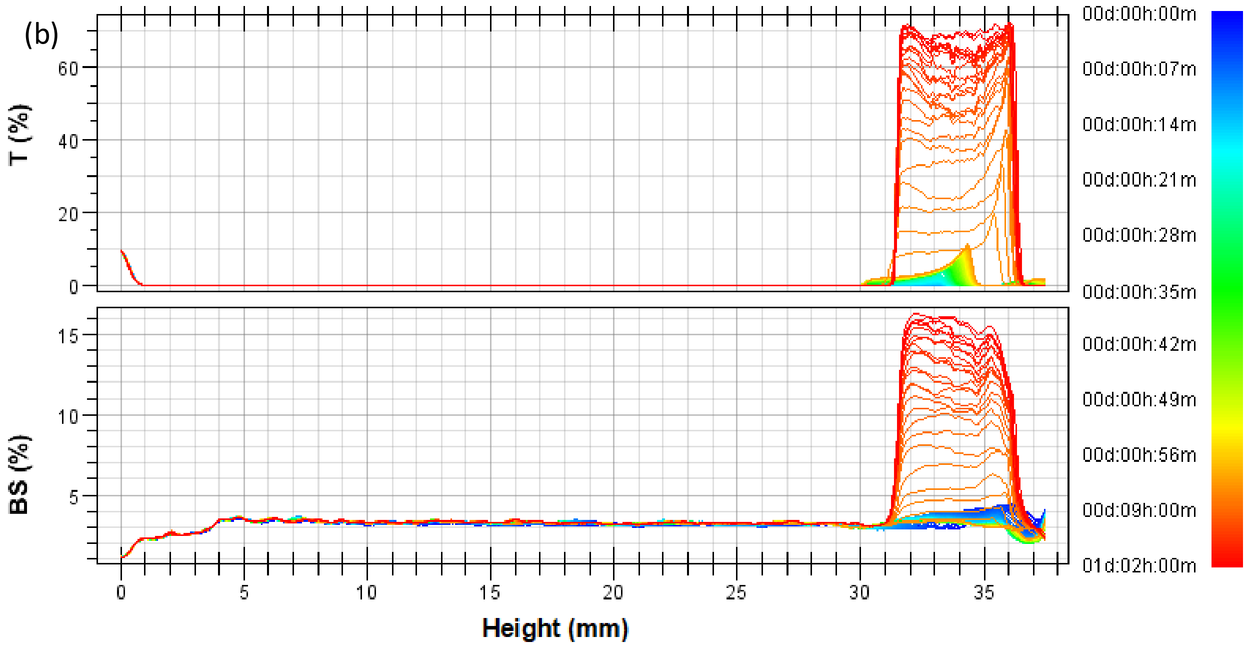
(b)
T (%)
BS (%)
Height (mm)
00d:00h:00m
00d:00h:07m
00d:00h:14m
00d:00h:21m
00d:00h:28m
00d:00h:35m
00d:00h:42m
00d:00h:49m
00d:00h:56m
00d:09h:00m
01d:02h:00m

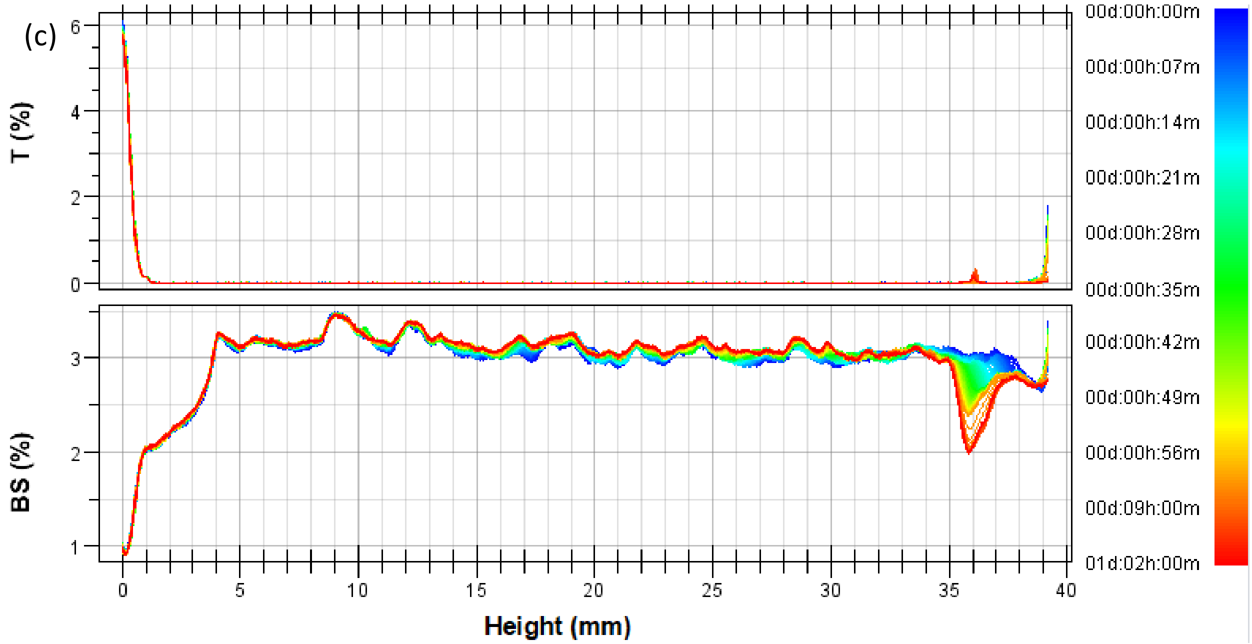
(c)
T (%)
BS (%)
Height (mm)
00d:00h:00m
00d:00h:07m
00d:00h:14m
00d:00h:21m
00d:00h:28m
00d:00h:35m
00d:00h:42m
00d:00h:49m
00d:00h:56m
00d:09h:00m
01d:02h:00m

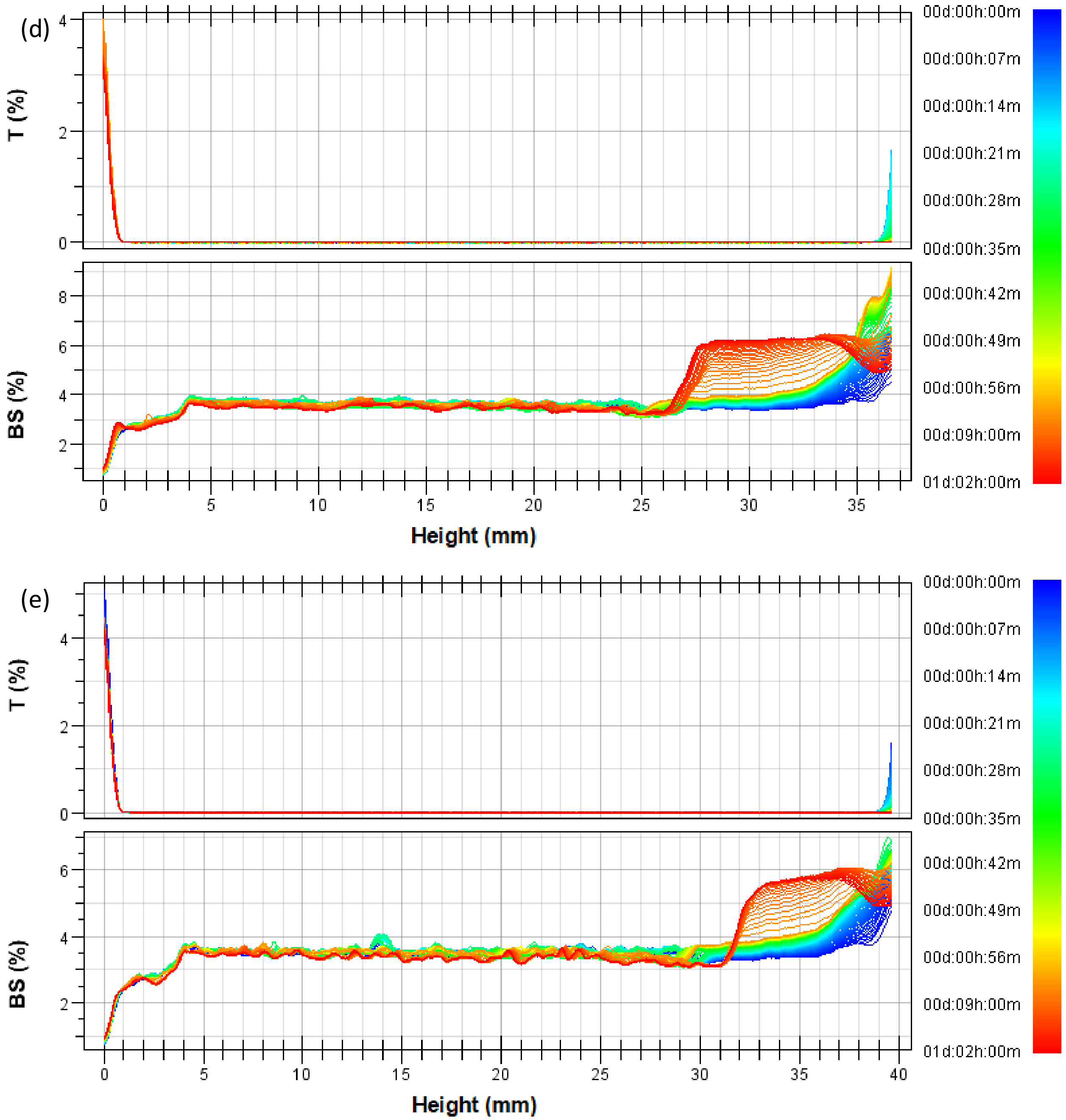

(d)
T (%)
BS (%)
Height (mm)
00d:00h:00m
00d:00h:07m
00d:00h:14m
00d:00h:21m
00d:00h:28m
00d:00h:35m
00d:00h:42m
00d:00h:49m
00d:00h:56m
00d:09h:00m
01d:02h:00m
(e)
T (%)
BS (%)
Height (mm)
00d:00h:00m
00d:00h:07m
00d:00h:14m
00d:00h:21m
00d:00h:28m
00d:00h:35m
00d:00h:42m
00d:00h:49m
00d:00h:56m
00d:09h:00m
01d:02h:00m

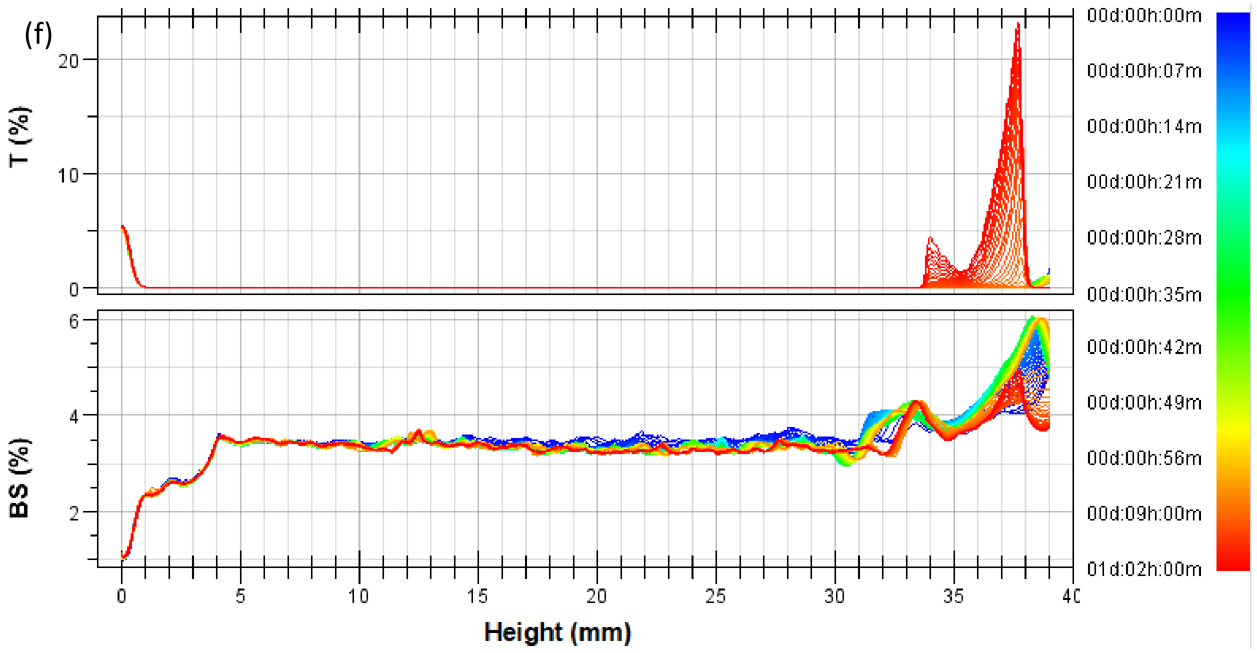

Fig. S1. Transmission (ΔT%) and backscattering (ΔBS) profiles at CL-1 dosages of a) 0, b) 0.05 wt. %, c) 0.10 wt.%, d) 0.25 wt.%~~.~~ a~~A~~nd transmission (ΔT%) and backscattering (ΔBS) profiles in the presence of e) CL-4 and f) CL-10 (at 0.1 wt.% dosage).
